# Diagnostic Accuracy of an Orofacial Scale for the Detection of Orofacial Myofunctional Disorders in Patients With Obstructive Sleep Apnoea

**DOI:** 10.1111/joor.13996

**Published:** 2025-05-01

**Authors:** Gislaine Aparecida Folha, Fabiana Cardoso Pereira Valera, Cláudia Maria de Felício

**Affiliations:** ^1^ Department of Health Sciences, Ribeirão Preto Medical School University of São Paulo Ribeirão Preto São Paulo Brazil; ^2^ Craniofacial Research Support Center University of São Paulo Ribeirão Preto São Paulo Brazil; ^3^ Department of Otorhinolaryngology, Ophthalmology, and Head and Neck Surgery, Ribeirão Preto Medical School University of São Paulo Ribeirão Preto São Paulo Brazil

**Keywords:** obstructive sleep apnoea, oral diagnosis, oral examination, stomatognathic system, validation studies

## Abstract

**Background:**

Patients with obstructive sleep apnoea (OSA) are known to have orofacial myofunctional disorders (OMD) compared to healthy individuals. Consequently, treatment with oropharyngeal exercises has shown promise in some OSA cases. However, this requires an adequate orofacial myofunctional evaluation.

**Aims:**

To develop a concise and reliable scale including the most relevant items for the orofacial myofunctional evaluation of individuals with OSA. The Expanded Orofacial Myofunctional Evaluation with Scores (OMES‐E) protocol was previously validated for this purpose. However, it has been criticised for its length, time‐consuming nature and omission of certain items.

**Materials & Methods:**

Fifty adults diagnosed with OSA by polysomnography and 23 healthy adults were included. Based on the literature, additional items were incorporated into the OMES‐E to enhance the evaluation (OMES‐E Plus), with subsequent reliability analysis. A scale with fewer items was derived from the OMES‐E Plus based on reliability and correlation analyses. Its accuracy, sensitivity and specificity were determined in a sample of 19 subjects with OSA and 19 without OSA (Control), matched for age and sex. Statistical analyses included Cronbach's alpha, item‐total correlations and receiver operating characteristic curve analysis.

**Results:**

The resulting new instrument, named the Orofacial Scale for Obstructive Sleep Apnoea (OFSOSA), comprises 31 items. The OFSOSA demonstrated significant discriminatory ability, with an area under the curve of 0.987, sensitivity of 95.5% and specificity of 93.7%.

**Conclusion:**

The OFSOSA, a concise instrument for myofunctional orofacial evaluation, was developed and validated, showing excellent psychometric properties for diagnosing OMDs in OSA patients.

## Graphical abstract

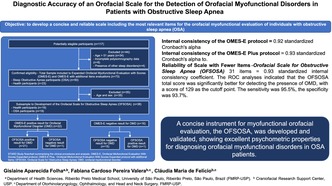

## 1 Introduction

Obstructive sleep apnoea (OSA) is a complex sleep‐related breathing disorder characterised by repetitive episodes of upper airway occlusion [1], involving a decrease or complete halt in airflow despite ongoing efforts to breathe. According to a recent systematic review and meta‐analysis of global studies [2], OSA is prevalent in approximately half of the worldwide population. Its association with cardiovascular diseases and increased mortality risk deserves attention.

There is evidence of airway remodelling in OSA patients, encompassing changes in sensation, muscle properties and neural drive [3]. With a multifactorial pathogenesis involving both ‘anatomical’ and ‘non‐anatomical’ factors, the potential role that elements beyond pharyngeal anatomy and craniofacial structure play in the pathophysiology of OSA has been recognised [4].

The muscles of the upper airways exhibit complex patterns of neural activation in response to different stimuli (mechanical or chemical), which vary among each muscle (e.g., the genioglossus, the largest pharyngeal dilator muscle located at the base of the tongue, and the tensor palatine muscle). The combination of loss of central drive and reflex input to upper airway muscles during sleep is believed to be an important contributor to the pathogenesis of OSA [5].

Several studies involving individuals with OSA have described changes in orofacial muscles and functions [6, 7, 8, 9, 10, 11, 12, 13], which can be referred to as orofacial myofunctional disorders (OMD). These are relatively common in the stomatognathic system, particularly among patients with sleep‐related respiratory disorders [6, 9].

Some clinical treatments for OSA have included oropharyngeal exercises, which have shown promising results [10, 13, 14, 15]. Consequently, there has been discussion regarding the potential of these exercises to address factors contributing to OSA in the orofacial and pharyngeal regions [11, 13].

Previously, due to the lack of a suitable protocol for the clinical evaluation of muscles and orofacial functions in individuals with OSA, the validity of the Expanded Orofacial Myofunctional Evaluation with Scores (OMES‐E) protocol [16] was assessed. It demonstrated good criterion validity and adequate construct validity, with the OSA group showing a worse orofacial myofunctional status compared to healthy subjects [9]. This finding has been confirmed by other studies [17, 18, 19]. Additionally, a recent study found that a higher body mass index (BMI) is associated with a greater prevalence of OSA, poorer sleep quality and more severe orofacial myofunctional conditions as measured by the OMES‐E protocol [20].

Despite the psychometric properties of the aforementioned protocol for orofacial myofunctional evaluation in OSA patients, one criticism is its lack of specificity in some items [11, 18]. In addition, the protocol is considered lengthy and time consuming for an initial examination.

Therefore, the objective of this study was to develop a concise and reliable scale based on the OMES‐E protocol, incorporating the most relevant items for individuals with OSA, to reduce the time required for evaluation and analysis. Additionally, the accuracy, sensitivity and specificity of the new scale were estimated.

We aspire for the new instrument to achieve the capability of accurately discriminating the orofacial myofunctional condition between individuals with OSA and healthy subjects, with a precision of 90% or higher.

## 2 Methods

In this retrospective study, the sample was selected from a database at the Craniofacial Research Support Center of the University of São Paulo, São Paulo, Brazil. The work was approved by the Human Research Ethics Committee of the Ribeirão Preto School of Medicine, University of São Paulo (São Paulo, Brazil) (approval number: 12634/2010), and all subjects provided written informed consent to participate. The study adhered to the Standards for Reporting Diagnostic Accuracy Studies (STARD) [21] to ensure completeness and transparency.

Data were collected from individuals aged 20 to 51 years who had been evaluated using the OMES‐E protocol and the additional items. In total, 73 individuals were selected, including 50 diagnosed with OSA and 23 healthy controls. The group with OSA was consecutively recruited from a sleep‐disordered breathing clinic. The control group consisted of individuals invited for convenience from the same community as the OSA group and met the eligibility criteria.

*Inclusion criteria for participants with OSA* included the following: previous otorhinolaryngologic evaluation (oroscopy, anterior rhinoscopy and nasofibroscopy) to confirm respiratory disorders and identify possible causes; snoring, as assessed by the Stanford Snoring Scale; daytime somnolence, measured using the Epworth Sleepiness Scale [22]; sleep efficiency > 75% and an apnoea/hypopnoea index (AHI) > 5 events/h during sleep, as determined by all‐night polysomnography (PSG) using the Biologic Sleep Scan VISION PSG* and following American Academy of Sleep Medicine parameters [23]; and no prior or current use of positive airway pressure (CPAP) devices or any intraoral devices for OSA symptom reduction.

*Inclusion criteria for healthy participants* were as follows: adequate sleep hygiene habits; no complaints of OSA when questioned by the examiner; no snoring according to the Stanford Snoring Scale; no daytime somnolence, as assessed by the Epworth Sleepiness Scale; and an otorhinolaryngologic evaluation (including oroscopy, anterior rhinoscopy and nasofibroscopy) confirming the absence of obstructive respiratory disorders.

Subjects with neurological or cognitive deficits, previous or current tumours or traumas in the head and neck region, current or prior orofacial myofunctional therapy, and/or current use of analgesics, psychiatric medications and muscle relaxants were excluded from the study.

### 2.1 Data Collection and Development of the New Instrument

#### 2.1.1 Examiners

Two speech‐language pathologists, who were previously trained and blinded to whether the subjects were healthy or OSA patients, confirmed intra‐ and inter‐examiner reliability and agreement values for the orofacial assessment [9]. The previous reliability coefficients for the assessments conducted with the OMES‐E by these examiners were 0.83 (intra‐examiner, test and retest) and 0.82 (inter‐examiner). The value of 0.80 demonstrated excellent agreement between examiners [9]. These coefficients were calculated by extracting data from the records for each examiner independently, twice, with no exchange of information between them and without access to their previous evaluations. A 15‐day interval was provided between the first and second rounds of assessments to avoid memory bias.

#### 2.1.2 Development of the Orofacial Scale for OSA (OFSOSA)

The content validity of the new instrument, which involved defining the object of interest and judging the relevance of each analysed variable, was established based on the OMES‐E protocol [9] and available literature on OSA [7, 10, 11]. Currently, only the OMES‐E protocol is validated for diagnosing OMD in patients with OSA.

The OMES‐E protocol was applied using previously described methodology [9]. This protocol allows for the evaluation of the components of the stomatognathic system in terms of appearance and posture (lips, tongue, face, cheeks, palate and the relationship between the maxilla and mandible), mobility (lips, tongue, jaw and cheeks) and functions (breathing, mastication and deglutition).

Predetermined scores were assigned, with the maximum scores attributed to normal patterns without deviation. The total sum of all scores in the protocol was 242.

Aligning with literature data on OSA, the following items, with the exception of lip sensitivity, were added to the OMES‐E protocol.

*Soft palate sensitivity:* Flexible monofilaments (SORRI Esthesiometer, Bauru, SP, Brazil) with six different thicknesses were utilised, with smaller filament thicknesses corresponding to lower amounts of force exerted in grams‐force (gf). The monofilament was positioned to touch the left side of the soft palate, within 1 cm from its origin in relation to the hard palate. A score of 4 was assigned when the subject identified the two filaments with the smallest diameters (0.07 and 0.2 gf), a score of 3 when identifying the two intermediate filaments (2.0 and 4.0 gf), a score of 2 when identifying the two filaments with the largest diameters (10.0 and 300 gf) and a score of 1 when the patient did not identify any monofilaments.

*Lip sensitivity*: The tactile sensitivity of this structure was assessed using the same methodology as in the soft palate tactile sensitivity evaluation, i.e., with flexible monofilaments. In this case, the monofilament was positioned on the central portion of the lower lip. This assessment was conducted because the lips, being more distant from the sites of OSA obstruction, can serve as useful control measures for assessing tactile sensitivity in more posterior oropharyngeal structures.

*Cheeks:* The presence (score 2) or absence (score 1) of dental markings on the buccal mucosa of the cheeks was verified.

*Soft palate morphology*: This assessment focused on length, with a score of 4 assigned for adequate length, 3 for slightly elongated, 2 for moderately elongated and 1 for severely elongated.

The ability to sustain voluntary contraction (endurance test) during tasks involving tongue contact with the palate, such as pressing the tongue body against the hard palate and tongue protrusion, was evaluated. For each task, a score of 4 was assigned when the subject maintained the contraction for 15 s or more without showing signs of fatigue (e.g., tremors, fasciculation, asymmetry in movement or cyanosis); a score of 3 was given for maintaining the contraction for 10–14 s; a score of 2 for 5–9 s; and a score of 1 when the subject was unable to perform the task or performed it for 4 s or less.

*Soft palate mobility*: This item was assessed through intermittent repetition of the vowel ‘a’ (‘a‐a‐a‐a‐a’), with the patient's performance evaluated on a 3‐point scale. A score of 3 was assigned when the subject performed the task normally, i.e., with precision and without difficulty; a score of 2 was given when the subject exhibited reduced mobility or showed signs of alteration, such as coughing or nausea, during the movement; and a score of 1 was assigned when the subject was unable to perform the task.

The following data were also obtained from each participant: sex and age; weight (kg), height (m), BMI (kg/m^2^) and cervical diameter (neck circumference in cm at the level of the cricoarytenoid joint). The data from this sample were analysed for specific purposes as follows.

### 2.2 Internal Consistency of the OMES‐E Protocol

Initially, the reliability and item analysis of the OMES‐E protocol (original) [8] was carried out to determine the degree of internal consistency using Cronbach's alpha, which measures the covariance among factors and ranges from 0 to 1 [18]. In this context, if all items (factors) measure the construct of myofunctional orofacial conditions, they should be highly related, resulting in a high alpha value. Due to the different amplitudes of the numerical scales of the protocol, the results were expressed as standardised Cronbach's alpha.

### 2.3 Internal Consistency of the OMES‐E Plus Protocol

Subsequently, to enhance the scale's precision in evaluating patients with OSA, the seven items described above were incorporated into the protocol. Once again, reliability was assessed.

### 2.4 Reliability of Scale With Fewer Items

In pursuit of developing a reliable scale with fewer items to distinguish the myofunctional conditions of patients with OSA from individuals without OSA symptoms, analyses were conducted starting with the OMES‐E Plus protocol.

Initially, items were excluded if the analysis indicated: (a) that removing an individual item could potentially increase the internal consistency of the scale (alpha if item deleted) and (b) a low correlation between the item and the total score with the respective item deleted (item‐total correlation, *r* < 0.38). Subsequently, further exclusions were made for items still meeting the criterion (b).

The resulting scale was named the *Orofacial Scale for Obstructive Sleep Apnoea* (OFSOSA) (see Supporting Information S1).

### 2.5 Accuracy, Sensitivity and Specificity of the Scales

For these analyses, a subsample of 2 groups with similar ages and sex distributions was extracted from the total sample: one group with OSA and another without OSA (control), each consisting of 19 participants. This choice was made to avoid the potential effects of these variables, especially age, on the orofacial myofunctional condition.

The sample size was determined prior to the study based on reported data on OMES‐E scores [9]. A minimum of 11 participants per group was required for statistical analysis with 80% statistical power (type II error, beta) and an alpha (type I error) set at 5%.

**FIGURE 1:**
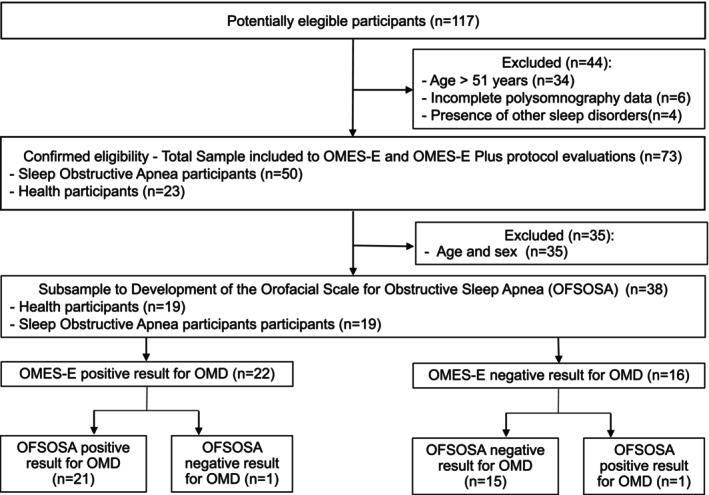
Flow of participants through study.

**FIGURE 2:**
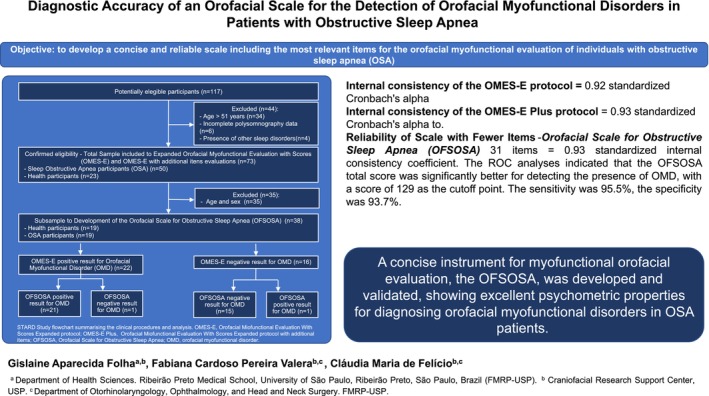
Study graphical abstract.

Using the OMES‐E as a reference standard and a cutoff point of < 186, the accuracy, cutoff point, sensitivity and specificity of the OFSOSA were determined. The total scores of the scales were considered for the analysis. Figure 1 shows the flow of patients through the study (Figure 2).

### 2.6 Statistical Analysis

A descriptive analysis was conducted to summarise the demographic and clinical data and the outcome scores. Student's *t*‐test was used for continuous data and the chi‐square test for categorical variables.

Cronbach's alpha, standardised Cronbach's alpha, the item‐total correlation and the average inter‐item correlation were determined using the reliability and item analysis method (Statistica software, version 14.0015, TIBCO Data Science, Palo Alto, CA, USA).

Receiver operating characteristic (ROC) curve analysis was used to determine the cutoff point, sensitivity and specificity of the OMES‐E and OFSOSA (MedCalc Statistical Software, version 22.023, MedCalc Software Ltd., Ostend, Belgium). The significance level was set at *p* < 0.05.

## 3 Results

Data from 73 subjects, 50 diagnosed with OSA (AHI median interquartile range [IQR] 28.90 [17.20–72.30]) and 23 healthy individuals without OSA symptoms were included based on specific criteria (Table 1).

**TABLE 1:** Distribution of sex, age, body mass index (BMI) and total evaluation scores in the total study sample.

*N* = 73	Control (*n* = 23)	OSA (*n* = 50)	*p*
Male sex	9 (39%)	31 (62%)	0.07^a^
Age (years)	30.43 (8.33)	40.14 (8.10)	0.0001^b^
BMI	23.47 (3.80)	31.93 (6.27)	0.0001^b^
OMES‐E score	198.95 (13.64)	163.34 (19.31)	< 0.0001^b^
OMES‐E Plus score	220.09 (15.01)	179.92 (20.45)	< 0.0001^b^
OFSOSA score	141.43 (10.94)	110.58 (16.84)	< 0.0001^b^

*Note:* Data represented as mean and standard deviation, except for sex for the total study sample.

Abbreviations: F: female, M: male, OFSOSA: Orofacial Scale for Obstructive Sleep Apnoea, OMES‐E Plus: OMES‐E protocol with additional items, OMES‐E: Expanded Orofacial Myofunctional Evaluation with Scores Protocol.

^a^ *p*‐value for chi‐square test.

^b^ *p*‐value for Student's *t*‐test.

The OMES‐E, composed of 44 items, demonstrated excellent internal consistency reliability, with a standardised Cronbach's alpha of 0.92.

The OMES‐E Plus, which consisted of 51 items, also demonstrated internal consistency reliability, with a slight increase in the standardised Cronbach's alpha to 0.93.

In this scale (51 items), the following items met both criteria adopted for exclusion (increased alpha if the item was deleted and item‐total correlation with *r* < 0.38 between the item and the total score with the respective item deleted): lip sensitivity, markings on the cheek mucosa, hard palate width, tongue retrusion movement, movements of elevation, lowering and protrusion of the mandible, and food bite. Additionally, the items face symmetry, proportion of facial thirds, nasolabial sulcus, deviation of the mandibular midline, lip volume, tongue position, hard palate height, tongue protrusion movement, tongue coupling to the palate, other behaviours suggestive of alterations in swallowing, and liquid and solid swallowing efficiency met the criterion of low item‐total correlation.

The resulting scale comprised 31 items. The standardised internal consistency coefficient was 0.93, and the item‐total correlation ranged from 0.40 to 0.69.

The OFSOSA result is obtained by summing all scores, with a maximum possible score of 161 representing the best orofacial myofunctional condition and a minimum possible score of 33 representing the worst condition.

### 3.1 Accuracy, Sensitivity and Specificity of the OFSOSA

Table 2 shows the demographic and clinical data, along with the total scores of the OMES‐E protocol and OFSOSA for 38 participants, who were divided into control (*N* = 19) and OSA (*N* = 19, AHI median IQR 25.21 [11.80–72.30]).

**TABLE 2:** Distribution of sex, age, body mass index (BMI) and total scores for the subsample.

*N* = 38	Control (*n* = 19)	OSA (*n* = 19)	*p*
Male sex, *n* (%)	8 (42.10)	11 (57.89)	0.52^a^
Age (years), median (IQR)	28.0 (26.2:35.0)	32.0 (26.5:37.0)	0.38^b^
BMI, median (IQR)	22.65 (21.0:25.3)	33.21 (29.8:35.9)	0.0001^b^
OMES‐E	199.79 (13.91)	166.05 (20.53)	< 0.0001^c^
OFSOSA	141.79 (10.86)	112.74 (16.57)	< 0.0001^c^

*Note:* In this table, a subsample of two groups with similar age and sex distributions is presented, extracted from the total sample for the analysis of accuracy, sensitivity, and specificity of the scales.

Abbreviations: IQR: interquartile range, OFSOSA: Orofacial Scale for Obstructive Sleep Apnoea, OMES‐E: Expanded Orofacial Myofunctional Evaluation with Scores Protocol.

^a^ *p*‐value for chi‐square test.

^b^ *p*‐value for Mann–Whitney test.

^c^ *p*‐value for Student's *t*‐test.

The ROC analyses indicated that the OFSOSA total score (sum of 31 items) was significantly better than chance for detecting the presence of OMD (AUC = 0.99, *p* < 0.001, 95% CI: 0.88%, 100%), with a score of 129 as the cutoff point. The sensitivity was 95.5% (95% CI: 77.2%, 99.9%), while the specificity was 93.7% (95% CI: 69.8%, 99.8%).

By using the cutoff value, relevant OMDs were present in all participants of the OSA group and in three participants from the control group.

## 4 Discussion

The aim of this study was to develop a reliable, concise and easy‐to‐use instrument for evaluating OMD in patients with OSA. This objective was achieved with the new OFSOSA, which includes items that best measure OMD in this population, demonstrating good internal consistency, item correlation and excellent psychometric qualities, thereby confirming the initial hypothesis of this study.

The use of OMD assessments, which enhance the reliability of results by adhering to standardised instructions and applying quantitative measures, has been recommended in recent years [24]. In a context where oropharyngeal exercises have shown promise as a treatment for OSA [7, 10, 13, 14], the OMES‐E [9] was the only published validated protocol for orofacial myofunctional evaluation in this population [11]. The present study also evidenced its excellent internal consistency reliability.

The OMES‐E Plus, which included additional items that could provide useful information about patients with OSA, showed a slightly higher reliability coefficient than the original protocol. This was expected, as adding more items to a scale designed to measure a specific concept (in this case, orofacial myofunctional condition) generally results in a higher degree of measurement reliability [25].

The OFSOSA, derived from the OMES‐E Plus protocol, emerges as a new evaluation tool that also adheres to the principles of psychophysical measurement. Despite having fewer items [26], the OFSOSA achieved very high internal consistency and accuracy in diagnosing OMD. In addition, it offers the advantage of quick and easy application, making it suitable for busy clinical settings or large study cohorts where the rapid completion of assessments is essential [3].

By assessing the stomatognathic system from different perspectives (morphological and physiological), the OFSOSA contributes to understanding the orofacial myofunctional condition of individuals with OSA. Sensory impairments play a fundamental role in upper airway obstructions, which are also significantly influenced by neuromuscular and anatomical alterations [3, 4].

The evaluation of the tongue in patients with OSA is crucial for determining individualised treatments, as it comprises the major dilator muscle of the upper airway and plays a critical role in OSA [27]. Regarding the newly introduced item, tongue endurance involves a high‐intensity task that requires sustained muscular contraction over time. This challenge arises from the predominance of type II fibres (fast‐twitch) in the tongue musculature, which are characterised by quicker contraction but faster fatigue compared to type I fibres (slow‐twitch). Thus, tongue endurance contrasts with tongue fatigue, where prolonged maintenance of steady muscle contractions indicates reduced susceptibility to fatigue in the tongue musculature [5]. Its assessment is crucial in both clinical and research settings, as it serves as an indicator of muscular performance, revealing the capacity of lingual muscles to sustain functional activities over time and providing valuable insights into orofacial muscular functionality. In the context of sleep disorders and breathing, weakened lingual muscles can negatively impact the upper airways, contributing to nocturnal obstructions and respiratory complications [28].

Furthermore, tongue endurance is significant in the realms of rehabilitation and therapy, particularly in the development of tailored exercise regimens. Its role extends to the prevention of complications and the systematic monitoring of progress within rehabilitation or intervention programs. In the study by Poncin et al. [29], enhancements in tongue endurance were observed among individuals with OSA following a specific therapeutic regimen. This improvement potentially contributed to the reduction of symptoms associated with OSA in that study. However, the authors highlighted the need for further research to determine the relationship between tongue endurance training and the restoration of the proportion of fast‐ and slow‐twitch muscle fibres. Thus, additional studies are imperative to clarify how specific tongue tasks might induce structural alterations in the upper airways, potentially reducing the severity of OSA.

The inclusion of the soft palate assessment ensued subsequent to comprehensive analyses. Soft palate mobility and length have been subjects of inquiry in clinical investigations [11]. These items allow for the consideration of physiological and morphological aspects in an awake state, factors that are relevant during the sleep cycle [11]. Notably, one study using computed tomography (CT) imaging [30] revealed an association between the measurement of soft palate length and velopharyngeal collapse in individuals affected by OSA.

The main limitation in this study was the lack of polysomnographic evaluation in the control group (healthy group). This decision was based on the absence of complaints related to snoring or excessive daytime somnolence, standardised scale scores [22] equal to or close to zero, and BMI values within normal limits. Consequently, the major clinical manifestations of the disorder [31] and the obesity‐related risk factors were not present, rendering the participants ineligible to meet the minimum criteria for PSG referral, as stipulated by Kushida et al. [26]. It is noteworthy that other studies involving control subjects [17, 18, 32] also did not perform polysomnographic evaluation. However, we acknowledge that clinical impressions or group categorisations based solely on symptoms lack the requisite accuracy for the precise diagnosis of sleep disorders.

The excellent psychometric values found add credibility to the application of OFSOSA in adult patients. Future studies may explore its validity in the paediatric population as well, given that oral‐facial anatomical problems play a fundamental role in the development of OSA in children [33].

The use of OFSOSA does not preclude the need for a more detailed myofunctional assessment in subjects who score below the cutoff found for the healthy group (C). However, the identified psychometric qualities could enhance the precision in recommending supplementary evaluations, therapeutic indications for oropharyngeal exercises, monitoring treatment outcomes and determining the appropriate time for discharge when used in conjunction with polysomnographic assessment results.

## 5 Conclusion

An instrument for myofunctional orofacial evaluation, the OFSOSA, has been developed and validated. This tool demonstrates excellent psychometric qualities, allowing for brief and accurate application in patients with OSA for the diagnosis of OMD.

## Author Contributions

**Gislaine Aparecida Folha:** conceptualisation, methodology, investigation, writing – original draft preparation, and review and editing. **Fabiana Cardoso Pereira Valera:** resources, writing – review and editing. **Cláudia Maria de Felício:** conceptualisation, methodology, formal analysis, writing – original draft preparation, and review and editing.

## Disclosure

The authors have nothing to report.

## Ethics Statement

The human subject research was approved by the Human Research Ethics Committee of the Ribeirão Preto School of Medicine, University of São Paulo (São Paulo, Brazil) (approval number: 12634/2010).

## Conflicts of Interest

The authors declare no conflicts of interest.

## Peer Review

The peer review history for this article is available at https://www.webofscience.com/api/gateway/wos/peer‐review/10.1111/joor.13996.

## Supporting information


Data S1.

## Data Availability

The data that support the findings of this study are available from the corresponding author upon reasonable request.
