# Supplementary material for: Diagnostic Accuracy of an Orofacial Scale for the Detection of Orofacial Myofunctional Disorders in Patients With Obstructive Sleep Apnoea
Source: J Oral Rehabil. 2025 May 1;52(9):1343–50. doi: 10.1111/joor.13996 (PMC12408965; doi:10.1111/joor.13996)
Supplement: Supplementary file 1 — Data S1. [file JOOR-52-1343-s001.docx]

**Diagnostic Accuracy of an Orofacial Scale for the Detection of Orofacial Myofunctional Disorders in Patients with Obstructive Sleep Apnea**

Gislaine Aparecida Folha^a,b^

Fabiana Cardoso Pereira Valera^b,c^

Cláudia Maria de Felício^b,c^

^a^ Department of Health Sciences. Ribeirão Preto Medical School, University of São Paulo, Ribeirão Preto, São Paulo, Brazil.

^b^ Craniofacial Research Support Center, University of São Paulo, Ribeirão Preto, São Paulo, Brazil.

^c^ Department of Otorhinolaryngology, Ophthalmology, and Head and Neck Surgery. Ribeirão Preto Medical School, University of São Paulo, Ribeirão Preto, São Paulo, Brazil.

Corresponding author:

Gislaine Aparecida Folha

Department of Health Sciences, 120 Miguel Covian Ave., USP Campus, Tel.: +55 (16) 3315-4417, E-mail: gislainefolha@fmrp.usp.br.

**Orofacial Scale for Obstructive Sleep Apnea (OFSOSA)**

| Date:______/_______/______   Age:__________      Birthday: ____/_____/____ |
| --- |
| Name:____________________________________________________________________ |
| Adress: ___________________________________________________________________ |

| **Lips Resting function** | | | |  | **Scores** |
| --- | --- | --- | --- | --- | --- |
| Closure with no apparent muscles contraction | | | | Normal | 4 |
| Dysfunction: Closure with effort or no labial closure | | | | Light | 3 |
|  |  |  |  | Moderate | 2 |
|  |  |  |  | Severe | 1 |
| **Labial commissures** | | | |  |  |
| At the level of the rima of the mouth and symmetric | | | | Normal | 4 |
| Dysfunction: Below of the rima of the mouth and/or asymmetrics | | | | Light | 3 |
|  |  |  |  | Moderate | 2 |
|  |  |  |  | Severe | 1 |
| Side below the rima of the mouth | right | left | both |  |  |
| **Mentalis muscle** | | | |  | **Scores** |
| Contraction not apparent (*with lips closure*) | | | | Normal | 4 |
| Dysfunction: contraction apparent | | | | Light | 3 |
|  |  |  |  | Moderate | 2 |
|  |  |  |  | Severe | 1 |

| **Lips mobility** | | Horizontal | | Lateral | |
| --- | --- | --- | --- | --- | --- |
|  | | Protrusion | Retrusion | To right | To left |
| Normal: precise movement |  | 6 | 6 | 6 | 6 |
| Insufficient ability (IA) |  | 5 | 5 | 5 | 5 |
| IA with associated movements |  | 4 | 4 | 4 | 4 |
| IA with tremor |  | 3 | 3 | 3 | 3 |
| IA with associated movement and tremor |  | 2 | 2 | 2 | 2 |
| Task no performed |  | 1 | 1 | 1 | 1 |

| **Cheeks** | | | |  | | **Scores** |
| --- | --- | --- | --- | --- | --- | --- |
| **Volume** | | | | Normal | | 4 |
| Dysfunction: Asymmetry between right and left sides | | | | Light | | 3 |
|  |  |  |  | Moderate | | 2 |
|  |  |  |  | Severe | | 1 |
| Increased | right | left | both |  |  | |
| Decreased | right | left | both |  |  | |
| **Tension/configuration** | | | | Normal | | 4 |
| Dysfunction: Flaccid / Drooping | | | | Light | | 3 |
|  |  |  |  | Moderate | | 2 |
|  | | | | Severe | | 1 |

| **Cheeks mobility** |  | To inflate | To suck | To retract | To transfer the air from right to left |
| --- | --- | --- | --- | --- | --- |
| Normal: precise movement |  | 6 | 6 | 6 | 6 |
| Insufficient ability (IA) |  | 5 | 5 | 5 | 5 |
| IA and associated movements |  | 4 | 4 | 4 | 4 |
| IA with tremor |  | 3 | 3 | 3 | 3 |
| IA associated movement and tremor |  | 2 | 2 | 2 | 2 |
| Task no performed |  | 1 | 1 | 1 | 1 |

| **Maxillo-mandibular relationships** |  | **Scores** |
| --- | --- | --- |
| **Vertical:** mandibular posture with free way space | Normal | 4 |
| Dysfunction: Teeth in occlusion without free way space |  | 3 |
| Without apparent tension | Light | 2 |
| With apparent tension | Moderate | 1 |
| With apparent tension | Severe | 4 |
| Dysfunction: Open mouth (> 4 mm) | Light | 3 |
|  | Moderate | 2 |
|  | Severe | 1 |
| **Anteroposterior** |  |  |
| Normal (overjet positive: 2±2) | Normal | 4 |
| Dysfunction: Maxilla Protrusion or Increased overjet | Light | 3 |
|  | Moderate | 2 |
|  | Severe | 1 |
| Dysfunction: Mandible Protrusion or Negative overjet | Light | 3 |
|  | Moderate | 2 |
|  | Severe | 1 |

| **Mandible mobility** | Right laterality | Left laterality |
| --- | --- | --- |
| Normal: precise movement | 6 | 6 |
| Insufficient ability (IA) | 5 | 5 |
| IA with associated movements | 4 | 4 |
| IA with tremor | 3 | 3 |
| IA with associated movement and tremor | 2 | 2 |
| Task no performed | 1 | 1 |

| **Tongue: volume/size** |  | **Scores** |
| --- | --- | --- |
| Compatible with the oral cavity | Normal | 4 |
| Dysfunction: Increased and/or widened | Light | 3 |
|  | Moderate | 2 |
|  | Severe | 1 |

| **Tongue mobility** | Horizontal | | Lateral | | Vertical | | Endurance |
| --- | --- | --- | --- | --- | --- | --- | --- |
|  | Protrusion | Retrusion | To right | To left | Raising | Lowering | Protrusion |
| Normal: precise movement | 6 | 6 | 6 | 6 | 6 | 6 | 6 |
| Insufficient ability (IA) | 5 | 5 | 5 | 5 | 5 | 5 | 5 |
| IA with associated movements | 4 | 4 | 4 | 4 | 4 | 4 | 4 |
| IA with tremors | 3 | 3 | 3 | 3 | 3 | 3 | 3 |
| IA with associated movement and tremor | 2 | 2 | 2 | 2 | 2 | 2 | 2 |
| Task no performed | 1 | 1 | 1 | 1 | 1 | 1 | 1 |

| **Soft palate** |  | **Scores** |
| --- | --- | --- |
| **Morphology** | Normal | 4 |
| Dysfunction: Long and / or Flaccid | Light | 3 |
|  | Moderate | 2 |
|  | Severe | 1 |
| **Sensibility** |  |  |
| 0.07gf or 0.2 gf filaments | Normal | 4 |
| 2.0 gf or 4.0 gf filaments | Light | 3 |
| 10.0 gf and 300 gf filaments | Moderate | 2 |
| did not identify any monofilament | Severe | 1 |
| **Mobility:** moviment raising (vowel /a/ produced intermittently /a a a/) | |  |
| Normal: precise movement | | 3 |
| Insufficient ability | | 2 |
| Absence of ability: task no performed | | 1 |

***Functions***

| **Breathing** (mode) |  | **Scores** |
| --- | --- | --- |
| Nasal breathing | Normal | 4 |
| Dysfunction: Mouth breathing | Light | 3 |
|  | Moderate | 2 |
|  | Severe | 1 |

| **Swallow: Lips behavior** |  | **Scores** |
| --- | --- | --- |
| Lips closure | Without effort | 4 |
| Dysfunction: Lips closure with effort (Presence of lips function, but with increased activity of lips and mentalis muscle) or absence of lips closure | Light | 3 |
|  | Moderate | 2 |
|  | Severe | 1 |

| **Swallow: tongue behavior** |  | | | **Scores** |
| --- | --- | --- | --- | --- |
| Contained in the oral cavity | Attached to the palate, without pressing against the dental arches | | | 4 |
| Between dental archs (or alveolar margins) | At limit of the incisal surfaces, with reduced vertical dimension of occlusion (VDO) | | | 3 |
|  | At limit of the incisal surfaces, with normal VDO | | | 2 |
|  | Exceeds the incisal surfaces and/or vestibular cusps | | | 1 |
| Interposition place | right | left | both |  |
|  | anterior | posterior | total |  |

| **Masticatory type** | According to chew strokes distribution | | | **Scores** |
| --- | --- | --- | --- | --- |
| Bilateral | Alternate (Chews stroke occurring 50% of the times on each side of the oral cavity, or 40% on one side and 60% on the other) | | | 10 |
|  | Simultaneous chews on both sides | | | 8 |
| Unilateral | Preference-grade 1 – (61% to 77% of the times on the same side) | | | 6 |
|  | Preference-grade 2 – (78% to 94% the times on the same side ) | | | 4 |
|  | Chronic (95% or more of the time on the same side) | | | 2 |
|  | Prefered/Chronic side | right | left |  |
| Anterior | Masticatory strokes occurring in the region of the incisors and canines | | | 2 |
| Function not performed | People did not chew | | | 1 |
